# The economic cost of bovine trypanosomosis in pastoral and ago pastoral communities surrounding Murchision Falls National park, Buliisa district, Uganda

**DOI:** 10.1186/s12917-022-03468-1

**Published:** 2022-10-17

**Authors:** Daniel Kizza, Michael Ocaido, Anthony Mugisha, Rose Azuba, Sylvia Nalubwama, Sarah Nalule, Howard Onyuth, Simon Peter Musinguzi, Charles Waiswa

**Affiliations:** 1 Department of Livestock and Industrial Resources, College of Veterinary Medicine Animal Resources and Biosecurity, Makerere University, Kampala, Uganda; 2 Department of Wildlife, Aquatic and Animal Resources, College of Veterinary Medicine Animal Resources and Biosecurity, Makerere University, Kampala, Uganda; 3 Faculty of Agriculture Department of Agricultural Production, Kyambogo University, Kampala, Uganda; 4 Department of Veterinary Pharmacy, Clinical and Comparative Medicine, College of Veterinary Medicine Animal Resources and Biosecurity, Makerere University, Kampala, Uganda

**Keywords:** Economic cost, Mortality loss, Milk loss, Bovine trypanosomosis, Buliisa

## Abstract

**Background:**

Animal diseases that are endemic like tsetse transmitted trypanosomosis cause the continuous expenditure of financial resources of livestock farmers and loss of productivity of livestock. Estimating the cost of controlling animal trypanosomosis can provide evidence for priority setting and targeting cost-effective control strategies.

**Methodology:**

A cross-sectional survey to estimate the economic cost of bovine trypanosomosis was conducted in cattle-keeping communities living around Murchision falls National Park, in Buliisa district Uganda. Data was collected on herd structure, the cost of treatment and control, prevalence of morbidity and mortality rates due to trypanosomosis, and salvage sales losses in cattle herds in the last year.

**Results:**

In this study, 55.4% (n = 87) of the households reported their cattle had been affected by trypanosomosis during the previous last year. There was a high economic cost of trypanosomosis (USD 653) per household in cattle-keeping communities in Buliisa district of which 83% and 9% were due to mortality and milk loss respectively/ High mortality loss was due to low investment in treatment. The study showed that prophylactic treatment 3 times a year of the whole herd of cattle using Samorin ® (Isometamidium chloride) at a cost of USD 110 could drastically reduce cattle mortality loss due to trypanosomosis due to trypanosomosis with a return on investment of USD 540 annually per herd. This could be coupled with strategic restricted insecticide spraying of cattle with deltamethrin products.

**Conclusion:**

The results show a high economic cost of trypanosomosis in cattle-keeping communities in Buliisa district, with cattle mortality contributing the largest proportion of the economic cost. The high mortality loss was due to low investment in treatment of sick cattle.

## Background of study

Animal trypanosomosis is one of the major limitations of cattle production causing a huge threat to household food security and livelihoods in sub-Saharan Africa. The disease impedes economic development and causes a huge toll on human health [1, 2]. The disease is majorly controlled using trypanocidal drugs or through control measures targeting the tsetse fly. In addition, the disease can be controlled by reducing the birthrate of disease vector through sterile insect technique and increasing the death rate of the disease vector through insecticide-treated cattle and insecticide impregnated traps and targets [3].

The effect of Animal African trypanosomosis (AAT) can be reduced through the use of curative and prophylactic trypanocides and rearing of trypanotolerant cattle [4]. Nevertheless, there are cases of increasing resistance to trypanocides and farmers are reluctant to rear trypanotolerant cattle [5].

There are several promising initiatives on vaccine candidates to control animal trypanosomes but currently, no vaccines are yet available for farmer use [6, 7].

The most suitable methods for controlling AAT and the magnitude to which they could be implemented depend on several factors, including social, economic, political, and environmental contexts. In addition, knowledge of the epidemiological cycle of AAT and the tsetse fly population and the available resources play a key role in control programs [5].

Although there have been several campaigns supported by international organizations to control AAT, decisions on allocation of resources have always been a challenge due to the large geographical range of the disease, the variation of the ecological and livestock systems and diversity of disease, and presence of different control methods [6, 7].

The control of livestock diseases including AAT is a private good where farmers have to pay for the service. For farmers to continuously invest in controlling Animal trypanosomosis the service must be affordable and effective [8, 9].

At the moment economic analysis of animal health has not been thoroughly studied [13–16]. Several reasons are contributing to few economic analysis studies on animal health and these include: (i) the complex impact of animal diseases - the direct effects of the diseases are easy to quantify while the indirect effects are difficult to approach; (ii) the complexity of livestock systems compared to crop systems due to inter alia to longer cycles and (iii) livestock systems are an integral part component of mixed farm systems [11, 12].

The control strategies targeting tsetse flies that have been deployed in Uganda include ground and aerial spraying of the breeding sites of tsetse, insecticide-treated cattle, and insecticide-impregnated traps and targets [10]. The use of these control measures has led to environmental toxicity and the high costs involved [16]. In Uganda, there are limited studies [13–17] where animal disease control decisions are based on economic cost. As such evaluation of the economic cost of tsetse and trypanosomiasis is necessary for deciding on the best cost-effective intervention strategy [18].

It is against this background that this study was designed to determine the economic cost of bovine trypanosomosis in Buliisa district Uganda.

## Materials and methods

**Fig. 1:**
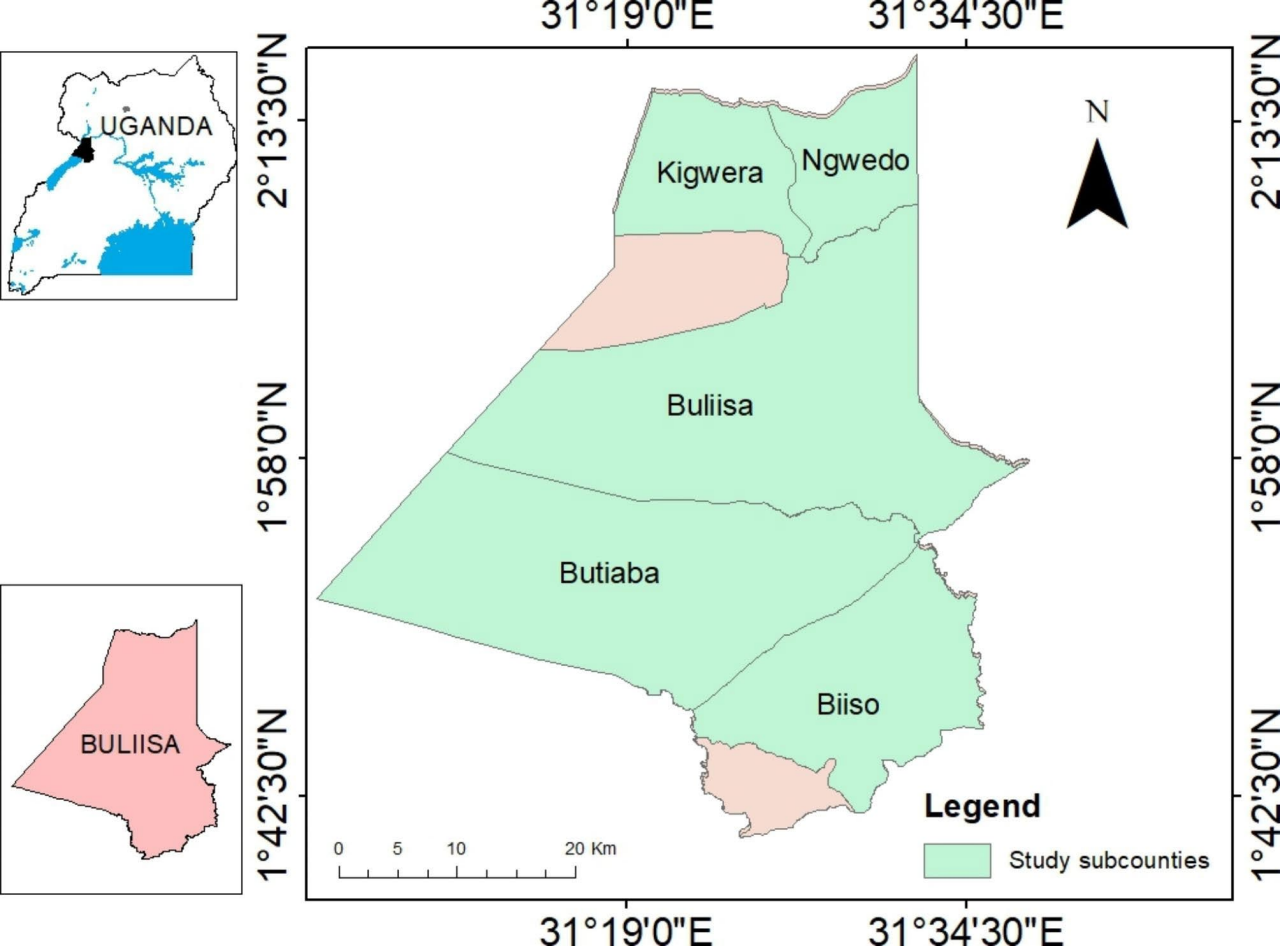
Map of Uganda and location of Buliisa district Source: Author

The study was conducted in Buliisa district located at (02º 11ʹ N 31º 24ʹ E) neighbouring Murchison Falls National Park. Details of the location were shown in Fig. 1. The choice of Buliisa district was based on its proximity to a national park and a higher prevalence of bovine trypanosomosis of 29.6%. The district is located in the cattle corridor belt bordering Nebbi district in North West, Nwoya district in North East, Masindi district in the East and Hoima district in the south, and Lake Albert in the West. Bugungu wildlife reserve which is part of Murchison Falls National park is located in Buliisa district. The district is rural-based with pastoralism, agro-pastoralism, fishing, and subsistence agriculture as the major economic activities. Buliisa experiences a bimodal type of climate with 2 rainy seasons (March to May and August to November). The vegetation is classified into forest, savannah, grassland, and swamp. The forest vegetation includes Budongo forest while savannah vegetation comprises perennial grasses, scattered trees, and shrubs. Murchison Falls National Park and Bugungu Game reserve contribute to grassland and woodland cover. Buliisa district is part of the Albertine graben where oil and gas have been discovered and explorations currently going on. The discovery of oil and gas has contributed to increased human activity and several infrastructural developments and employment opportunities for both local and foreign workers. Buliisa district has 6 sub counties and 1 town council. These include Biiso, Buliisa, Kihungya, Butiaba, Kigwera, Ngwedo and Buliisa town council. The sub counties are further sub divided into parishes and several villages.

A cross-sectional survey was conducted from January to April 2020 using a pre-tested structured questionnaire. Data was collected from 157 participants that were randomly selected. The selection criteria of study participants were being a cattle farmer and voluntarily consenting to participate in the study. The participants were drawn from the list of cattle keepers provided by local leaders and veterinary extension staff in each sub county. Through the Coordinating Office for the Control of Trypanosomosis in Uganda (COCTU) focal person, Bullisa District Production Office (DPO), and District Veterinary Officer (DVO) were approached and explained the objectives of the study. The DVO contacted the sub-county Animal Husbandry Officers (AHO) who in turn were explained the study objectives and trained as research assistants. Sub counties of Biiso, Buliisa, Butiaba, Kigwera and Ngwedo were visited and study sites selected.

The questionnaire was pre-tested and additional information generated and some questions were modified. The questionnaire was translated from English into Runyoro by Makerere University Center for languages and communication services (CLCS).

The sample size for the study was computed using the following formula

$$n = \frac{N}{{1 + N{\varepsilon ^2}}}$$

Where n = minimum returned sample size, N = population size, ℇ= adjusted margin of error which is

$$\left[ {\varepsilon = \frac{{\rho e}}{t}} \right]$$

e = degree of accuracy expressed as a proportion (Margin of error at 0.03 for continuous data), ρ = number of standard deviation that would include all possible values in a range for a 5 point scale which is equal to 4, t = t value for selected alpha level = 1.96 at 95% confidential interval [19].

The questionnaire collected information on participants’ socio-demographic characteristics, crop and livestock enterprises, cattle herd structure, prices per each cattle category, and the number of cattle age category that was affected by trypanosomosis in the last year. Furthermore, additional information was collected on the cost of curative and prophylactic treatment which included drugs used, and the cost of insecticide used in controlling tsetse flies. The number of abortions in the cattle herd, mortality of animals due to trypanosomosis in the last year, and salvage sales of cattle in the last year were also collected. In addition, data was collected on how communities controlled tsetse flies.

Economic data was collected and collated from the questionnaires. Data was then coded and entered into Microsoft Excel® 2020 spreadsheet software which was used to generate descriptive analysis mainly presented as means and percentages. Herd cattle age mortality rates due to trypanosomosis during the last year were determined. Cattle that presented with common signs of trypanosomosis before their death was included and taken as trypanosomosis-induced mortality. Herd cattle age morbidity rates were calculated from cattle that presented common signs and symptoms of trypanosomosis during the last year.

Mortality loss was calculated by computing the number of age categories of cattle that died from trypanosomosis multiplied by the prevailing market price of that age category of cattle. Salvage value was calculated from the number of cattle that were infected with trypanosomosis and sold before they died at a salvage price for the last year.

Sales loss was computed as the difference between the normal sale value and the salvage value. The percentage price reduction was calculated as a ratio total salvage value to the total normal sale value multiplied by 100.

The economic cost due to bovine trypanosomosis was calculated as the sum of costs due to: (i) treatment and chemoprophylaxis of the disease in the herd; (ii) loss due to mortality; (iii) estimated loss of milk production from literature [20] due to lack of records. The estimation was based on the following assumptions (Lactation off take (liters per year) 280 * (Number of lactating cattle that died in previous year) 125* (Average price per liter in UG. shs.)1000 ; (iv) live animal salvage sale loss; (v) insecticide spraying costs; (vi) tsetse fly trap costs; (vi) bush clearing costs.

## Results

The average farm size was 29.8 ± 7.2 acres. On average the household had 32 ± 3.1 cattle, 10 ± 1.2 goats, 0.7 ± 0.17 pigs, 10 ± 1.5 sheep, and 14.5 ± 1.1 chicken.

The percentage of age-specific herd structures were shown in Table 1.

**Table 1:** Percentage of cattle herd structure

Cattle category	Percentage (%)
Lactating cattle	26.3
Dry cattle	20.5
Heifers	15.4
Steers	4.4
Weaners	9.2
Female calves	11.3
Male calves	9.7
Bulls	3.2

In this study, 55.4% (n = 87) reported their cattle had been infected by trypanosomosis during the previous year. Annual expenditure on treatment using Samorin® (Isometamidium Chloride was Ug Shs. 12,147 (USD 3.47) per household. In addition, 74% of the households treated their cattle themselves without the supervision of veterinarians. The average cost of a sachet of Isometamidium chloride (Samorin ®) treating 8–10 cattle was at Ug. Shs. 30,000 or USD 8.5. Isometamidium Chloride was administered at an interval of 2–3 months a year. The mean prices of cattle per age-specific category were shown in Table 2. The age-specific morbidity and mortality rate were as shown in Table 3.

**Table 2:** Mean (Uganda shillings) per cattle age category

Cattle age category	Mean Price
Lactating cattle	957,727 ± 59,647
Dry cattle	901,075 ± 35,090
Heifers	707,647 ± 16,996
Weaners	503,158 ± 22,936
Steers	615,223 ± 66,561
Male calves	346,571 ± 19,132
Female calves	416,641 ± 33,046
Bull	1,300,946 ± 59,831

**Table 3:** Percentage Mortality and morbidity rates of cattle age categories due to trypanosomosis

Age category	Morbidity Rate	Mortality Rate
Lactating cows	20.0	8.3
Dry cows	90.7	5.5
Heifers	15.8	6.1
Weaners	28.2	8.6
Steers	36.7	17.7
Male calves	12.1	7.8
Female calves	12.1	8.6
Bulls	20.8	7.1
Overall	33.4	7.8

*Exchange rate 1 USD = 3500 Ug Shs. at the time the study was conducted*Milk loss was computed as (Lactation off take (liters per year) 280 * (Number of lactating cattle that died in previous year) 125* (Average price per liter in UG shs.)1000 .**Sales loss was computed as the difference between the normal sale value and the salvage value. The percentage price reduction was calculated as a ratio total salvage value to the total normal sale value multiplied by 100.**

74% of the households treated their cattle themselves without the supervision of veterinarians. The average cost of a sachet of Isometamidium Chloride (Samorin ®) was at Ug. Shs. 30,000. One sachet was used for treating 8–10 cattle. Generally no prophylactic of cattle was being done. To prophylactically protect cattle against bovine trypanosomosis cattle need to be treated 2–3 times a year with Samorin®. This would cost USD 110 per herd.

Cattle were not sprayed with insecticides against tsetse flies. Farmers who reported practicing bush clearing and bush burning were 10.2% and 3.2% respectively. The mean bush cleared area was 0.21 acres. The results further showed that 5% of households used tsetse traps as a control method for the tsetse flies.

## Discussion

The results from the study show that cattle was a major livestock species reared followed by indigenous chicken, goats, and sheep. This finding broadly supports the work of other studies that highlighted the role of cattle and other livestock species in supporting pastoralist livelihoods [20–22]. Cattle in pastoral and agro-pastoral communities play a multifunctional role in providing both market and non-market benefits. The latter include financing and insurance functions which define the competitiveness of cattle rearing in pastoral and agro-pastoral communities [23]. Cattle and other types of livestock in pastoralist and agro-pastoral households support an important role in coping with shocks, accumulating wealth, and acting as a bank in the absence of commercial financial institutions and formal markets. [24] .

In terms of cattle herd structure, adult cattle were the majority in household herds. Heifers, female calves, and weaners followed in that order (Table 1). The results show that more female cattle were kept compared to male calves and bulls. The findings might indicate that pastoralists keep more female cattle because of their ability to produce milk and for herd growth. This finding is consistent with another study [25] where female cattle of reproductive age constituted more than 50% of all livestock species. This is contrary in areas where male cattle are used for traction.

The overall prevalence and mortality rate of bovine trypanosomosis was 33.4% and 7.8% respectively (Table 3). The findings are not based on blood screening rather on cattle that presented with common signs of bovine trypanosomosis in the last one (prevalence) and before their death (trypanosomosis induced mortality). The findings are suggestive of prevalence and mortality farmers reported based on the clinical signs the animals presented since livestock disease diagnostic services are not available. Due to absence of laboratory diagnostic services in the district, the overall prevalence reported can also be attributed to other diseases presenting similar clinical signs to those of bovine trypanosomosis. These results are higher than those found in Metekel Zone North West Ethiopia which reported a prevalence of 12.1% and a mortality rate of 4.4% [24]. These differences in prevalence and mortality rates could be caused by variations in vegetation types and the seasons when the studies were conducted. The type of vegetation and season are known to determine the tsetse population and consequently the prevalence and mortality rates [25–27]. In addition, another plausible reason for the difference could be attributed to the breed of cattle kept. In areas where crossbred cattle are kept compared to indigenous breeds, it’s likely to find higher prevalence and mortality rates. From this study, the highest mortality rate was reported in the steer category of cattle while the highest morbidity rates were observed among dry cattle. A possible explanation for this might be that larger animals were more attractive to tsetse flies compared to smaller animals. Large cattle produce more odor plumes that attract tsetse than calves. This was further supported in previous studies [2] and [26].

The control measures of trypanosomosis mainly involved use of trypanocidal drugs with isometamidium chloride (Samorin®) as the main drug of choice. Although the drug is more expensive compared to other trypanocidal drugs on the market, farmers revealed that it has both curative and protective effects on animals. The farmers’ revelations were in support with a previous study [27] where it was reported that Isometamidium chloride mode of action was both therapeutic and prophylactic. The results from our analysis showed that 1 sachet of Samorin® costs Ug shs.30, 000 and farmers usually use it to treat 10 animals. When used in prophylaxis treatment at a three months interval (30,000/10)* 4 times a year, it would cost per herd of 32 animals (Average herd size) Ug shs. 384,000 or USD 110 annually per herd. This would drastically reduce the high mortality rate loss caused by trypanosomosis (Table 4) thereby increasing the profit margins of cattle keeping in the area. This was in agreement with studies.

**Table 4:** Mean annual economic cost in Ug. Shs. of Bovine trypanosomosis per household

Economic cost	Ug. shs	% contribution EC
Treatment	12,147	0.5
Mortality loss	2,057,073	83.0
Insecticide cost	80,210	3.2
Milk loss	222,930	9.0
Salvage sale loss	46,197	1.9
Bush clearing	6,739	0.3
**Total UGX**	**2,425,296**	
**USD**	**693**	

done elsewhere [28, 29] where they found higher returns on investment was got when farmers used trypanocide prophylaxis to protect their cattle against trypanosomosis.

In addition farmers in this area did not spray their cattle against tsetse flies using insecticides. In other areas infested with tsetse flies [11, 16] farmers have used dual-purpose insecticides like deltamethrin to control both ticks and tsetse with success. Spraying the entire animal’s body uses large amounts of the insecticide wash which is costly and leads to environmental contamination. The Restricted Insecticide Application protocol (RAP) is now being advocated for [30]. RAP involves application of insecticide to tsetse predilection sites of the animal (bellies, fore, and hind legs) and in the ears. These are also the predilection sites of *Rhipicephalus Appendiculatus*. The anticipated benefits of RAP compared to full body spraying include reduced over-dependence on trypanicidal drugs, lowered risk of drug resistance, and cost of tsetse and tick-borne disease control [16, 30, 31].

From this study (Table 5) it was shown that dry cattle and steers were salvage sold at a price less than market value. Salvage sales were done by farmers to avoid complete loss as a result of death. Animals that were salvaged sold are ones that failed to respond to treatment and continue deteriorating in their health till the farmer decides to dispose of them before dying. As a result, farmers made losses depending on the state of the animals and the salvage price offered. It was found that farmers lost 56.1% of their income due to salvage sales. This was far less compared to the percentage loss of 83% for bulls and 88% for cows caused by foot and mouth disease outbreak in Isingiro [32].

**Table 5:** Total (for all households in the study n-157) and mean household mortality and salvage sale loss

Age category	Mortality loss	Sale loss
Lactating cows	119,634,375	0
Dry cows	56,767,725	3,834,675
Heifers	37,505,291	577,647
Weaners	23,145,268	0
Steers	30,761,150	2,491,338
Male calves	14,902,553	0
Female calves	23,331,896	349,141
Bulls	16,912,246	0
Total (n = 157)	322,960,504	7,252,801
Average household loss	2,057,073	46,196

The mean annual economic cost per household due to trypanosomosis was found to be USD 693 of which 83% and 9% were due to mortality and milk loss respectively (Table 4). The mortality loss was equivalent to USD 588 which was higher than USD 244 reported in Metekel zone Ethiopia [33] and USD 200 in Baro Akobo and Gojeb river basins Ethiopia [33] There are several possible explanations for this result. One possible explanation might be that the mortality loss is contributed by other diseases that can present signs similar to those of trypanosomosis. However, in this area, there was a lack of laboratory services where farmers and field veterinarians can diagnose blood samples to confirm the presence of trypanosomes before treatment. This finding is in agreement with results of an earlier study [34] which reported that the use of veterinary diagnostic laboratories in Uganda was poor. Also, there were no veterinary diagnostic services found in the area. The farmers were treating cattle themselves failing to administer the right curative trypanocides at the right dose. There was therefore a need to provide trypanosomosis diagnostic and veterinary services for sick cattle. Also, there are substandard and fake trypanocidal drugs on the market which may have contributed to treatment failure.

The drive by most farmers to improve genetically their herds through crossbreeding may also have contributed to the high mortality in crossbred animals compared to local breeds [2, 35].

When farmers invest in a preventive prophylactic treatment using Samorin ® at an interval of every 3 months per year, the annual cost of treatment per household would be USD 110. The return on investment in treatment would be USD 465. This could be saved annually making cattle-keeping enterprise profitable venture in this area. This, therefore, means that a prophylactic treatment regime should be adopted in this area.

Milk loss of USD 63.4 annually per household due to trypanosomosis is the second largest contribution to the total economic cost. Milk loss was computed by multiplying number of lactating cattle that died in previous one year by the average price per liter and the estimated lactation offtake (liters per year) [36]. The loss in milk was mainly through death of lactating cows, abortions of dry cows, and decreased milk yield in sick cattle. Milk is an important component of the communities’ diet and milk loss undermines the daily household incomes. Milk that was not directly consumed was locally processed into other value added dairy products that could be sold locally. With increasing population in Buliisa district and the oil discovery within the district, the demand for milk is growing hence becoming a major source of household income.

Surprisingly, the percentage contribution of treatment and bush clearing is less than 1% (Table 4) yet more than 50% of the households reported their animals were infected with trypanosomosis the previous year. The small contribution of treatment cost to the total economic cost of trypanosomosis may be contributing to the high mortality loss observed in cattle due to trypanosomosis. In addition, most farmers keep local breeds of cattle that are thought to be more trypanotolerant and therefore are reluctant to invest in treatment costs compared to farmers with crossbreed animals which are have shown to be trypanosusceptible.

In this study, bush clearing and use of traps were not used by most farmers. A possible explanation for the low practice of bush clearing might be that land is communally owned and communities were not motivated to invest in it despite knowing that bushes were breeding habitats for tsetse. Bush of different types provides a good breeding environment for different tsetse species. The *Glossina palpalis* and G *fusca* tsetse species thrive well in woody vegetation while the *G*. *moristan* species survive best in savannah woodland. Furthermore, indiscriminate bush clearing as an approach to controlling the tsetse population can lead to a negative impact on biodiversity loss and the approach is not ecologically and politically acceptable. However, there has been modification developed [37, 38] which include removal of vegetation at ground level without removing high trees (discriminative partial bush clearing) or cutting only some of the trees or shrubs species (partial selective bush clearing) which are effective in reducing the tsetse populations. Traps were not being deployed as a tsetse control measure in the study area. The probable reason why traps are not popular among the farmers might be the lack of their promotion as an important tool to monitor spatial and temporal changes in the tsetse population and non-functional livestock extension, entomology, and community tsetse control intervention programs [39]. There are several limitations to the wider use of traps which could be non-community involvement in their deployment, supervision, and management, high cost, and high rate of theft and vandalism.

Relatedly bush or vegetation influences the efficiency of use of insecticide-impregnated traps and targets. The effectiveness of traps and targets in controlling tsetse flies can be hampered by vegetation regrowth and encroachment [40] found a significant decrease in tsetse catches when the traps were obscured by vegetation by 80%.

## Conclusion

The results show a high economic cost of trypanosomosis (USD 653) in cattle-keeping communities in Buliisa district with death of cattle contributing the largest proportion to economic cost (83%). Prophylactic treatment of cattle using Samorin® costing USD 110 annually could significantly reduce cattle mortality due to trypanosomosis with a net return on investment of USD 465 annually per herd.

## Recommendation

Prophylaxis treatment using Samorin® should be done three times a year. This should be coupled with community participation in strategic restricted spraying of cattle with deltamethrin products to control both tsetse flies and ticks.

## Data Availability

The dataset(s) supporting the conclusions of this article is (are) available from the corresponding author on reasonable request.

## Abbreviations

AHO: Animal Husbandry officer
AAT: Animal African Trypanosomosis
COCTU: Coordinating office of the control of trypanosomosis in Uganda
DPO: District Production officer
DVO: District Veterinary officer
CLCS: Center for Language and Communication services
RAP: Restricted insecticide application protocol
USD: United States dollar
Ug. Shs.: Uganda Shillings
